# Latrunculin A Treatment Prevents Abnormal Chromosome Segregation for Successful Development of Cloned Embryos

**DOI:** 10.1371/journal.pone.0078380

**Published:** 2013-10-24

**Authors:** Yukari Terashita, Kazuo Yamagata, Mikiko Tokoro, Fumiaki Itoi, Sayaka Wakayama, Chong Li, Eimei Sato, Kentaro Tanemura, Teruhiko Wakayama

**Affiliations:** 1 Laboratory for Genomic Reprogramming, Center for Developmental Biology, RIKEN, Kobe, Japan; 2 Laboratory of Animal Reproduction, Graduate School of Agricultural Science, Tohoku University, Sendai, Japan; 3 Center for genetic analysis of biological responses, Research Institute for Microbial Diseases, Osaka University, Suita, Japan; 4 Asada Institute for Reproductive Medicine, Asada Ladies Clinic, Kasugai, Japan; 5 School of Medicine, Tongji University, Shanghai, China; 6 Managing director, National Livestock Breeding Center, Nishishirakawa-gun, Japan; 7 Department of Biotechnology, Faculty of Life and Environmental Science, University of Yamanashi, Kofu, Japan; The University of Hong Kong, Hong Kong

## Abstract

Somatic cell nuclear transfer to an enucleated oocyte is used for reprogramming somatic cells with the aim of achieving totipotency, but most cloned embryos die in the uterus after transfer. While modifying epigenetic states of cloned embryos can improve their development, the production rate of cloned embryos can also be enhanced by changing other factors. It has already been shown that abnormal chromosome segregation (ACS) is a major cause of the developmental failure of cloned embryos and that Latrunculin A (LatA), an actin polymerization inhibitor, improves F-actin formation and birth rate of cloned embryos. Since F-actin is important for chromosome congression in embryos, here we examined the relation between ACS and F-actin in cloned embryos. Using LatA treatment, the occurrence of ACS decreased significantly whereas cloned embryo-specific epigenetic abnormalities such as dimethylation of histone H3 at lysine 9 (H3K9me2) could not be corrected. In contrast, when H3K9me2 was normalized using the G9a histone methyltransferase inhibitor BIX-01294, the *Magea2* gene—essential for normal development but never before expressed in cloned embryos—was expressed. However, this did not increase the cloning success rate. Thus, non-epigenetic factors also play an important role in determining the efficiency of mouse cloning.

## Introduction

The oocyte’s reprogramming ability to reset the genomic specialization of a somatic nucleus is the most efficient method used so far for cloning [1,2] and can give rise to full-term cloned animals [3,4]. Since cloned mice were first produced in 1998 [4], there have been many attempts at improving the birth rate [5], such as modifying the methodology [4,6], controlling the DNA acetylation status [7] and changing the timing of oocyte enucleation [8]. However, many disparities remained between normally fertilized embryos and cloned embryos and the birth rates of cloned embryos are still very low. The main cause of these problems is regarded as the incomplete reprogramming of the somatic epigenome and there have many studies aimed at improving the epigenetic status of cloned embryos [6,7,9-11]. However, some clone-specific epigenetic abnormalities, such as dimethylation of histone H3 at lysine 9 (H3K9me2), have never been corrected to the same level as in normally fertilized embryos by any treatment [12,13], whereas treatment with trichostatin A (TSA) improved the success rate of cloned mice by correcting other epigenetic abnormalities.

Here, we tried to improve the birth rate of cloned mice produced by somatic cell nuclear transfer (SCNT) in terms of alleviating both genetic and epigenetic problems in cloned embryos. For the epigenetic approach, we focused on H3K9me2, which serves as a binding region for heterochromatin protein 1 (HP1) [14,15]. HP1 localizes to heterochromatin domains [16] and is involved in downregulation of the *Magea* and *Xlr* family genes [10]. These genes have never been expressed in any cloned embryos even when genetically manipulated donor cells were used [6,10]. For the non-epigenetic approach, we focused on abnormal chromosome segregation (ACS). Although it is not clear whether ACS in cloned embryos is biologically or technically problem [17] and Balbach et al. reported that the karyotypes and chromosome segregation in cloned mouse embryos are not as bad either [18,19], at least some cloned embryos showed ACS and we recently reported that the birth rate of cloned mice could be improved by modifying the protocol rather than via epigenetic alteration [20]. Generally, reconstructed oocytes should be treated with actin polymerization inhibitors such as cytochalasin B (CB) or D during activation to keep all chromosomes inside the ooplasm, otherwise pseudo-second polar bodies can be extruded and some chromosomes derived from the donor nucleus will be lost [21]. Nevertheless, we found that F-actin localization in CB-treated cloned embryos was different from that seen in normally fertilized embryos. When we used latrunculin A (LatA)—a G-actin polymerization inhibitor—instead of CB, all chromosomes were kept inside the ooplasm without adverse effects on F-actin and the birth rate of cloned mice was increased [20]. Actin is an abundant protein present in all eukaryotic cells, and actin polymerization and depolymerization play fundamental roles in biological processes such as cell migration, determining cell shape, vesicle trafficking and regulating transcription [1,2,22,23]. It is also known that the F-actin meshwork that forms in the nuclear space is essential for preventing chromosome loss and aneuploidy in the embryo [24]. Such aneuploidy is one of the main causes of death in cloned embryos [25].

In the present study, we used live cell imaging to examine how LatA treatment affected the full-term development of cloned embryos not only in terms of epigenetic factors such as histone modifications and gene expression, but also for non-epigenetic factors such as chromosome segregation in early embryogenesis. In addition, we tried to correct H3K9me2 in cloned embryos using BIX-01294 (BIX) [26]. This is a specific inhibitor of the G9a histone methyltransferase responsible for dimethylation of H3K9 at transcriptionally silent regions [27,28]. However, this attempted correction of H3K9me2 did not improve the successful full-term development rate of cloned embryos.

## Materials and Methods

### Animals

B6D2F1 (C57BL/6 × DBA/2) strain female mice, aged 8–10 weeks, were used to produce oocytes. The surrogate pseudopregnant female mice used as embryo transfer recipients (see below) were ICR strain mice mated with vasectomized male mice of the same strain. B6D2F1 and ICR mice were purchased from Shizuoka Laboratory Animal Center (Hamamatsu, Japan). All animal experiments conformed to the Guide for the Care and Use of Laboratory Animals and were approved by the Institutional Committee of Laboratory Animal Experimentation of the RIKEN Center for Developmental Biology.

### Collection of oocytes

Mature oocytes were collected from the oviducts of 8–10-week-old female mice that had been induced to superovulate with 5 IU pregnant mare serum gonadotropin (Teikokuzoki, Tokyo, Japan) followed by 5 IU human chorionic gonadotropin (hCG, Teikokuzoki) 48 h later. Cumulus-oocyte complexes (COCs) were collected from the oviducts approximately 16 h after hCG injection. After collection, COCs were placed in HEPES-buffered CZB medium (H-CZB) [29] and treated with 0.1% bovine testicular hyaluronidase (Sigma-Aldrich, St Louis, MO, USA). After several minutes, the cumulus-free oocytes were washed twice and then cultured in a droplet of potassium simplex optimized medium (KSOM) (Millipore [U.K.] Ltd., Watford, UK) at 37 °C under 5% CO_2_ in air until used.

### ICSI

Spermatozoa were collected from the cauda epididymidis of a mature B6D2F1 male mouse and incubated in H-CZB medium for more than 30 min at room temperature. ICSI was performed as described [30]. Briefly, the sperm head was separated from the tail by applying several piezo pulses to the neck region and the head was then injected into an oocyte. After 10 min of recovery at room temperature, the oocytes were cultured in KSOM as above until used for experiments.

### In vitro fertilization

Cumulus-intact oocytes were collected in 0.2 ml of HTF medium [31] and inseminated with capacitated spermatozoa (final concentration 100/μl) [32]. After 2 h incubation at 37 °C under 5% CO_2_ in air, the cumulus cells were dispersed by pipetting and cultured in KSOM as above for 96 h.

### SCNT and embryo transfer

The SCNT procedure was performed as described [4]. Groups of oocytes were transferred into a droplet of H-CZB containing 5 μg/ml cytochalasin B (CB) for enucleation of the second meiotic division (MII) spindle. Oocytes undergoing microsurgery were held with a holding pipette and a hole was made in the zona pellucida following the application of several piezo pulses (Prime Tech, Ibaraki, Japan) to an enucleation pipette. The MII chromosome–spindle complex was aspirated into the pipette with a minimal volume of ooplasm. After enucleation of all oocytes, they were each injected with a cumulus cell nucleus. After nuclear transfer, the reconstructed oocytes were activated using 10 mM SrCl_2_ in KSOM with 2 mM EGTA [33] in the presence of 5 µg/ml CB for 6 h or 5 µM LatA for 10 h. In both group, 50 nM TSA treatment was continued for 10 h [11]. Activation time of 10 h is longer than usual method, but full term development of cloned embryos was not affected by longer treatment of SrCl_2_ [20]. In some experiments, 3 nM BIX was added together with TSA. Some cloned embryos were fixed for immunostaining and other cloned embryos were cultured in KSOM at 37 °C under 5% CO_2_ after three washes in KSOM. The preimplantation development rates of cloned embryos were examined from the PN to the blastocyst stages.

### Embryo transfer and examination of placentae

To produce placenta in the LatA and CB experiments, ICSI-generated and cloned embryos at the 2-cell stage were transferred into the oviducts of pseudopregnant ICR females at 0.5 days postcoitus (dpc) and placentas were collected by caesarean section at 19.5 dpc. Placental weights were measured at the time of caesarean section and the placentas were fixed with 4% paraformaldehyde (PFA) for paraffin wax embedding and histology. The placentae were sectioned for staining with hematoxylin and eosin. In the BIX treatment experiments, cloned embryos at the morula stage were transferred into the uteri of pseudopregnant ICR females at 2.5 dpc and the live offspring were collected by caesarean section at 19.5 dpc.

### Live cell imaging

Chromosomal dynamics during the first, second and third mitotic division of ICSI and cloned embryos were analyzed using live cell imaging technology. Messenger RNAs encoding EGFP–α-tubulin and mRFP–H2B were prepared as described [34]. Briefly, after linearization of the template plasmid at the Xba I site, mRNA was synthesized using the T7 RiboMAX^TM^ Large Scale RNA Production System (Promega, Madison, WI, USA). The 5′ end of each mRNA was capped using Ribo m7G Cap Analog (Promega). To circumvent the integration of template DNA into the embryo genome, reaction mixtures from in vitro transcription runs were treated with RQ-1 RNase-free DNase I (Promega). Synthesized RNAs were treated with RQ-1 RNase-free DNase I (Promega). Synthesized RNAs were treated with phenol/chloroform followed by ethanol precipitation. After dissolution in RNase-free water, mRNAs were subjected to gel filtration using a MicroSpin^TM^ G-25 column (Amersham Biosciences, Piscataway, NJ, USA) to remove unreacted substrates and then stored at -80 °C until used. Microinjection of mRNAs into oocytes was performed as described [35]. ICSI-generated or reconstructed oocytes were transferred to droplets of H-CZB medium in the observation chamber and a few picoliters of mRNA solution were introduced into the oocyte cytoplasm using a piezo-activated micromanipulator with a glass micropipette (1–3 μm diameter). The embryos were transferred to 5 μl drops of CZB medium on a glass-bottomed dish and placed in an incubation chamber set at 37 °C on the microscope stage. A gas mixture of 5% CO_2_ and 95% air was introduced into the chamber. Fifty-one images in the z-axis and two color images were captured at 15 min intervals using a live cell imaging system [34]. Device control and image analysis were performed using MetaMorph software (Molecular Devices, Sunnyvale, CA, USA).

### Immunostaining

Embryos at 10 h after activation were fixed in PBS containing 4% paraformaldehyde for 30 min. The fixed oocytes were washed twice in PBS containing 1% (w/v) BSA (Nacalai Tesque, Kyoto, Japan) (PBS-BSA) for 15 min each and then stored in PBS-BSA containing 0.1% (v/v) Triton X-100 (Nacalai Tesque) overnight at 4 °C. Embryos were then incubated with the primary antibodies: rabbit polyclonal anti-acH3K9 (1:100 dilution, Upstate Biotechnology Inc., Lake Placid, NY, USA), rabbit polyclonal anti-acH3K14 (1:100 dilution; Upstate Biotechnology Inc.), rabbit polyclonal anti-H3K9me2 (1:200 dilution; Millipore, Billerica, MA, USA), rabbit monoclonal anti-H3K4me2 (1:100 dilution; Abcam Japan, Tokyo, Japan) or goat polyclonal anti-heterochromatin protein (HP) 1α (1:100; Santa Cruz Biochemicals, Santa Cruz, CA, USA) and mouse monoclonal anti-H2B (1:400 dilution; Abcam Japan) in PBS-BSA overnight at 4 °C. After the embryos had been washed twice in PBS-BSA for 15 min each, they were incubated for 1 h with dye-conjugated secondary antibodies: Alexa-Fluor 488-labeled goat anti-mouse IgG (Molecular Probes Inc., Eugene, OR) and Alexa-Fluor 546-labeled goat anti-rabbit IgG (Molecular Probes Inc.). After the embryos had been washed twice in PBS-BSA, the DNA in embryos not stained with the anti-H2B antibody was stained with 2 μg/ml of DAPI (Molecular Probes Inc.). Then, after the embryos had been washed twice in PBS-BSA, serial images were taken using fluorescence confocal microscopy (FV-1000, Olympus Corp., Tokyo, Japan). Relative levels of acH3K9, acH3K14, H3K9me2, H3K4me2 and HP1α in embryos were measured using Olympus Fluor View (Olympus). Embryos were examined in PBS at 25 °C.

### Real-time RT–PCR

Complementary DNA sequences from single ICSI-generated or cloned blastocysts were synthesized using Arcturus PicoPure RNA Isolation kits (Life Technologies, Carlsbad, CA, USA). Quantitative PCR was carried out on a StepOnePlus Real-Time RT–PCR system (Applied Biosystems, Foster City, CA, USA) using Fast SYBR Green Master Mix (Applied Biosystems). The *Gapdh* gene in each embryo was used for endogenous reference. The data on gene expression levels were analyzed using StepOnePlus software (Applied Biosystems). Primer sequences were as follows: 5′-gctccaactcctctgacctg-3′ and 5′-tgtccaatgagggtacagca-3′ for *Magea2*; 5′-agcagaattcaaggcaggag-3′ and 5′-gtccatctcaaccagccaat-3′ for *Xlr5c*; and 5′-ttcaccaccatggagaaggc-3′ and 5′-cccttttggctccaccct-3′ for *Gapdh*.

### Statistical analysis

The incidence of ACS, blastocyst formation rates and offspring birth rates were evaluated using Chi-squared tests. Fluorescence levels, gene expression levels, and placental weights were analyzed by ANOVA followed by Fisher’s protected least significant difference test; P < 0.05 was assumed to be statistically significant.

## Results

### Abnormal chromosomal segregation (ACS) during early embryogenesis

**Figure 1:**
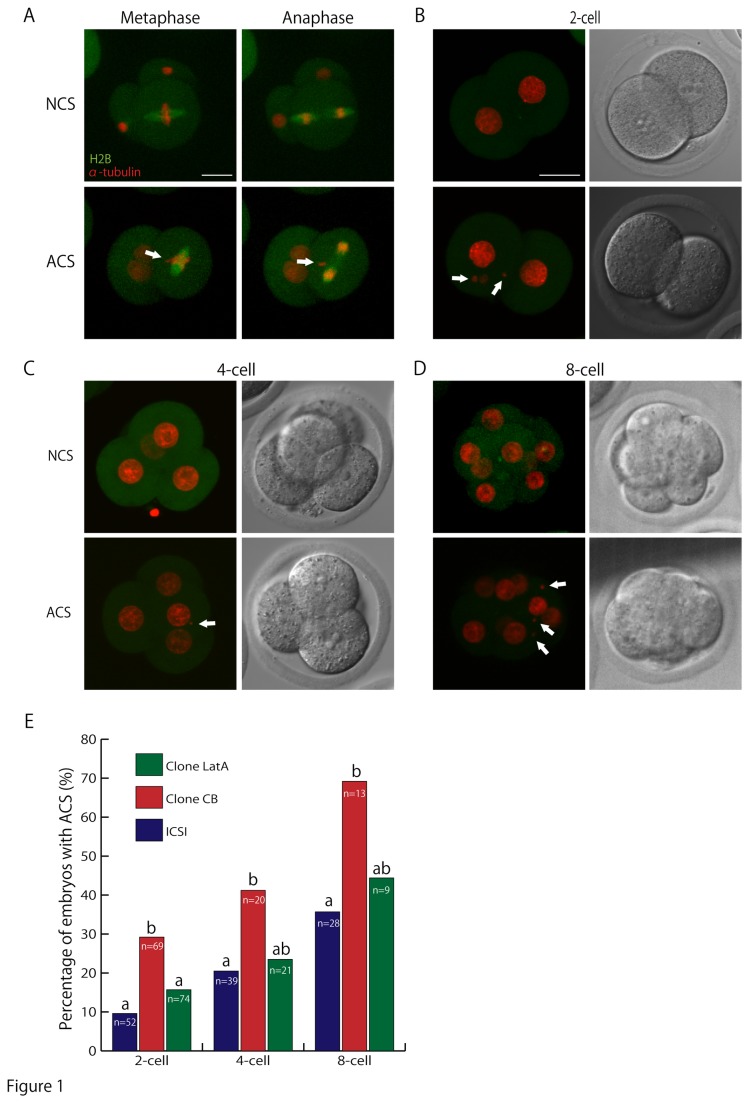
Chromosome segregation in early embryonic development. (A) Chromosomes were misaligned at metaphase and lagging chromosomes were found at anaphase in embryos ACS was occured. (B-D) Merged and bright field images of embryos with normal chromosomal segregation (NCS) and abnormal chromosomal segregation (ACS). As an example, time-lapse images of chromosome segregation at the first, second and third mitosis are shown at the 2-cell, 4-cell and 8-cell stages, respectively. Arrows indicate chromosomal fragments appearing during the division. Green, EGFP-α-tubulin; red, mRFP–H2B. Bar = 30 µm. (E) The percentages of embryos with ACS at the 2-cell, 4-cell and 8-cell stages in ICSI-generated and cloned embryos. Values with different superscripts in the same category differ significantly between ICSI-generated, CB- and LatA-treated cloned embryos.

In a previous study, we reported that abnormal F-actin localization in cloned embryos was corrected by using LatA treatment [20]. Among many roles of F-actin in the cytoplasm, here we focused on chromosomal segregation from the 1-cell to the 8-cell stage, which is critical for postimplantation development. Monomeric red fluorescent protein coupled with histone H2B (mRFP–H2B) and EGFP-tagged α-tubulin were expressed in embryos by mRNA injection immediately after SCNT or intracytoplasmic sperm injection (ICSI) and chromosomal segregation was monitored. Some embryos at these stages showed abnormal chromosomal behavior in mitotic blastomeres. Parts of the chromosomes were misaligned with the metaphase plate and lagging chromosomes were found at anaphase (Figure 1A). Consequently, ectopic micronuclei appeared as a result of ACS (Figure 1B-D). The incidence of ACS in cloned embryos produced with CB treatment was significantly higher than in ICSI-generated control embryos at the first, second and third cell division. However, when using LatA instead of CB, the incidence of ACS decreased significantly at the 2-cell stage and became similar to control embryos at the 4-cell and 8-cell stages (Figure 1E).

### Epigenetic abnormalities in cloned embryos produced with CB or LatA

**Figure 2:**
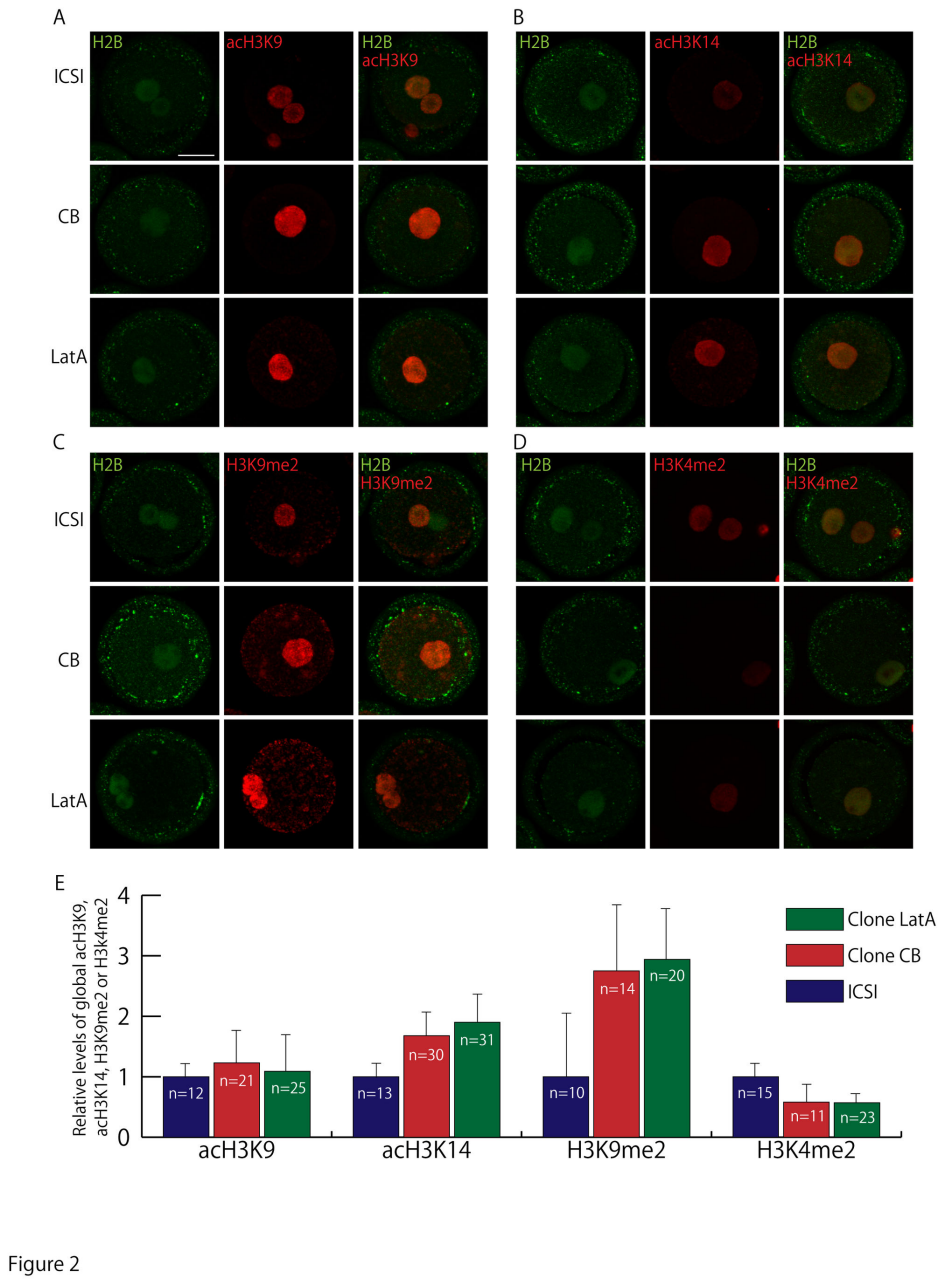
Histone modifications in 1-cell stage embryos. (A-D) acH3K9, acH3K14, H3K9me2 or H3K4me2 levels in ICSI-generated and CB- or LatA-treated cloned embryos. Bar = 30 µm. (E) The intensities of immunofluorescence for acH3K9, acH3K14, H3K9me2 and H3K4me2 relative to that of H2B. They are compared with the intensities in ICSI-generated control embryos. The acetylation or methylation levels of these regions were not different between LatA- and CB-treated cloned embryos.

Our next question was whether the increased success rate of cloned mice produced by LatA treatment arose from modifications of epigenetic abnormalities. To investigate this, CB- or LatA-treated cloned embryos and ICSI-generated embryos were fixed at the pronuclear stage and immunostained with antibodies to acetylated histone H3 at lysines 9 or 14 (acH3K9 or acH3K14, respectively), to H3K9me2, to dimethylated histone H3 at lysine 14 (H3K14me2) and to H2B. As shown in Figure 2, the acetylation or methylation levels were not different between CB- and LatA-treated cloned embryos, but were abnormally high or low, respectively, compared with control embryos.

### Epigenetic abnormalities in cloned placentae produced with CB or LatA treatments

**Figure 3:**
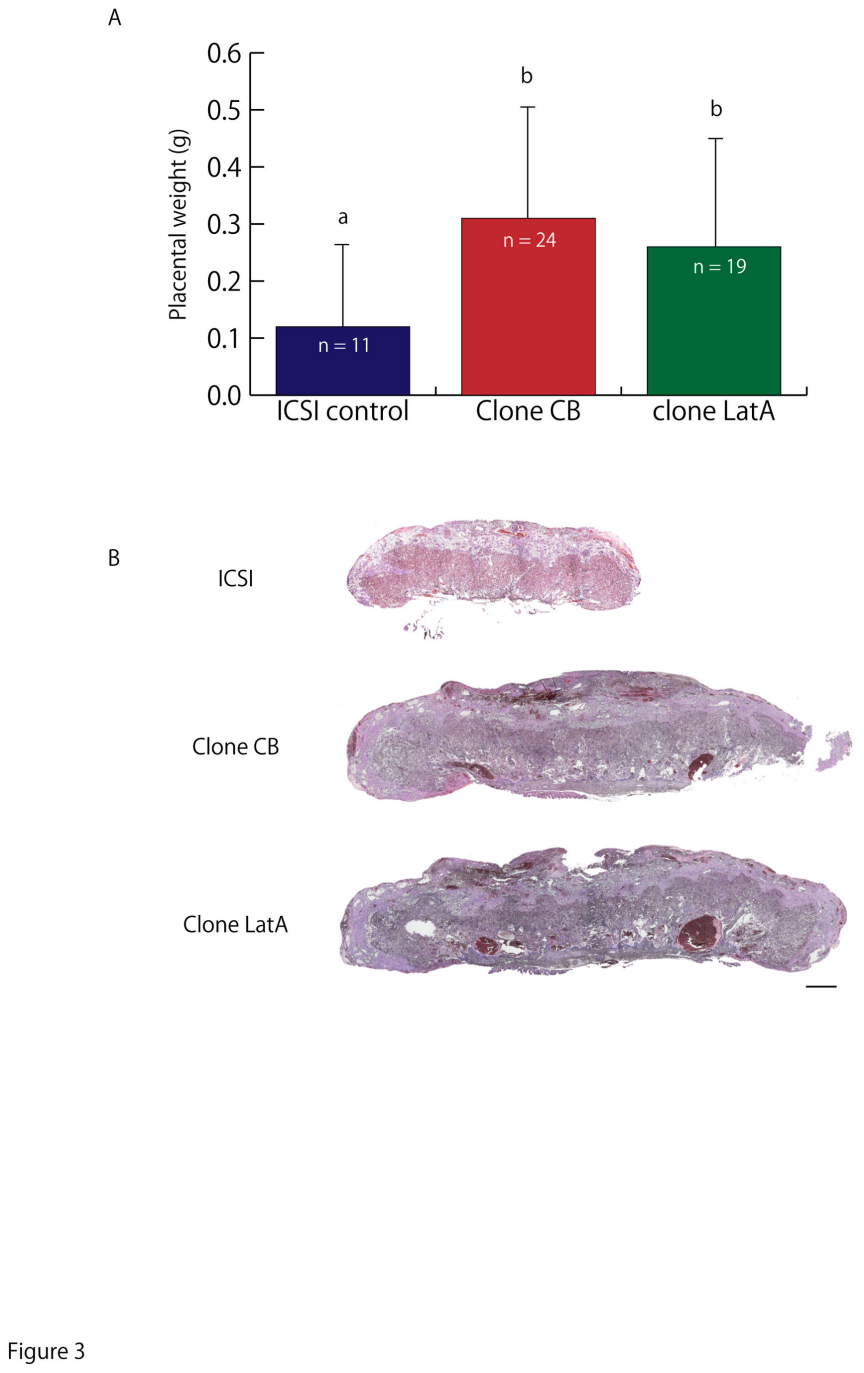
Abnormalities in placentae derived from cloned embryos. (A) Placental weights from ICSI-derived control embryos and CB- or LatA-treated cloned embryos. Error bars indicate SD. Asterisks indicate significant difference at p < 0.05. (B) Hematoxylin and eosin staining of placentae derived from ICSI-derived control and CB- or LatA-treated cloned embryos. Abnormal distortion of the boundary between the spongiotrophoblast and labyrinth layers was observed in placentas derived from both CB- and LatA-treated cloned embryos. Bar = 500 μm.

It is well known that most cloned animals possess abnormal placentae arising from epigenetic errors [36-38]. Placentae derived from cloned or ICSI-generated control embryos were collected at embryonic day (E)19.5 and their weights and histology were compared. Abnormally heavy placentae and the abnormal distortion of the boundary between spongiotrophoblast and labyrinth layers were not corrected with LatA treatment (Figure 3).

### Effect of BIX-01294 on cloned preimplantation embryos

**Figure 4:**
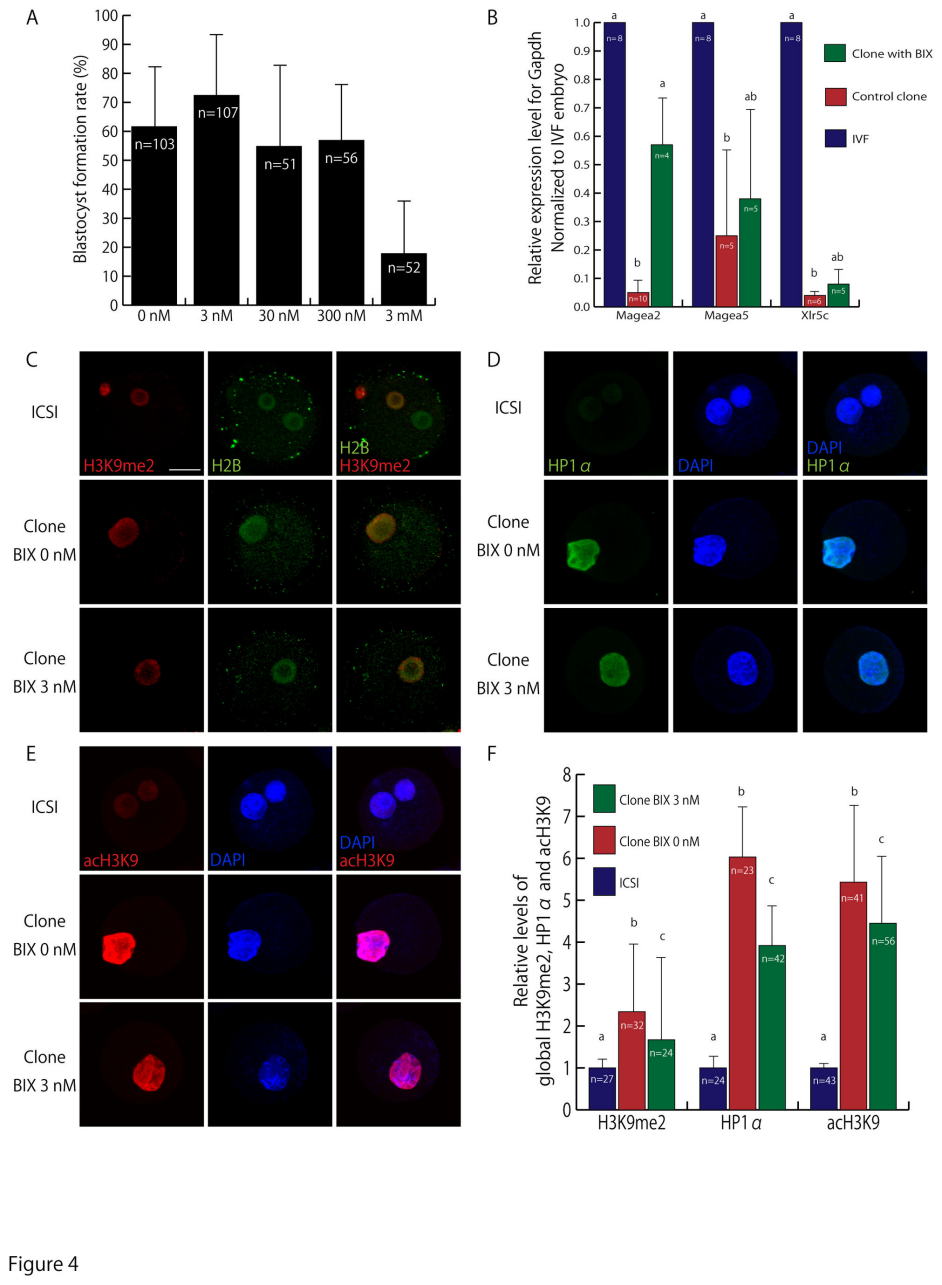
The effect of BIX on cloned embryos. (A) Blastocyst formation rate of cloned embryos treated with various concentrations of BIX. (B) Relative expression level of *Magea* and *Xlr* family genes in a blastocyst. The level of mRNA was normalized to the *Gapdh* expression level in the same sample. These genes were strongly repressed in control cloned blastocysts, but the reduced *Magea2* expression was increased by treatment with BIX. (C–F) Fluorescence level of H3K9me2, HP1α and acH3K9 normalized to the DAPI or histone H2B level of the same sample. Bar = 30 μm. BIX treatment was effective in decreasing H3K9me2, HP1α and acH3K9 levels.

H3K9me2 plays an important role in gene silencing but so far no cloning method has been able to correct abnormally high H3K9me levels in cloned embryos. We tried to control the H3K9me2 status in cloned embryos by using BIX, a specific inhibitor of the G9a histone methyltransferase. First, we examined the maximum concentration of BIX that did not decrease the blastocyst formation rate in control embryos and decided to use 3 nM, which led to the highest blastocyst formation rate. (Figure 4A). Then we examined the effect of this treatment on cloned embryos in terms of G9a target gene expression and H3K9me2 level. Real-time RT–PCR was performed at the blastocyst stage using ICSI-generated, control cloned embryos and BIX-treated cloned embryos. As shown in Figure 4B, *Magea2* gene expression was significantly upregulated by BIX treatment (P < 0.05). This result is consistent with the report that *Magea2* expression in ES cells is controlled by G9a through H3K9me2 at the promoter site [26]. When 1-cell stage cloned embryos were examined by immunostaining, BIX treatment decreased the H3K9me2, HP1α and acH3K9 levels significantly (Figure 4C–F; P < 0.05).

### Effects of BIX on full-term development of cloned embryos

Finally, we examined whether correcting the epigenetic abnormalities at H3K9me2 in cloned embryos could lead to full-term development. Although BIX treatment reduced the level of H3K9me2 in cloned preimplantation embryos, the birth rate of cloned mice following BIX treatment was 7.8% (8/102), almost the same as untreated controls (7.3%, 9/123; Table 1). Thus, alleviating this particular epigenetic aberration in cloned embryos did not improve development to term.

**Table 1:** The effect of BIX on full term development of cloned embryos.

Treatment	No. reconstructed oocytes	No. survived after activation	No. PN formed	No. 2-cell embryos (%/PN)	No. morulae (%/PN)	No. embryos transferred (recipients)	No. live offspring (%/ET)	Mean body weight (g)	Mean placental weight (g)
Control	239	178	160	146 (91.3)	123 (76.9)	123 (13)	9 (7.3)	1.80 ± 0.24	0.30 ± 0.09
BIX 3 nM	194	152	140	125 (89.3)	102 (72.9)	102 (8)	8 (7.8)	2.12 ± 0.43	0.33 ± 0.04

PN, pronuclei; ET, embryo transfer

## Discussion

Previously, we reported that LatA treatment corrected aberrant F-actin localization in cloned embryos and increased the birth rate of cloned mice [20]. However, the association between inhibition of actin polymerization at the 1-cell stage and full-term development was not clear. F-actin in the nucleus has an important role in chromosome transport [24] and cloned embryos exhibiting ACS before the 8-cell stage cannot develop to full term [25]. We confirmed here that the occurrence of ACS was significantly higher than controls in CB-treated cloned embryos at the 2-cell stage, but this decreased significantly to the same level as ICSI-generated embryos when using LatA instead of CB. Thus, LatA appears to improve the birth rate associated with ACS in cloned embryos by gently inhibiting actin polymerization; importantly, this event was not linked directly to epigenetic alterations. When specific histone modifications associated with either active (acH3K9 and H3K4me2) or repressed (H3K9me2) chromatin, and with aberrant spindles in oocytes (acH3K14) [27,28] were examined, these were not improved by using LatA instead of CB.

Next, we focused on the H3K9me2 abnormality in cloned embryos because its level decreases quickly in normally fertilized embryos to allow the proper expression of several genes but persists in cloned embryos [6,12,13,39-42]. This methylated histone region serves as a binding platform for HP1 [14,15,43]; correspondingly, HP1 expression was significantly higher in cloned embryos than in zygotes fertilized in vitro [44]. In addition, H3K9-related genes, such as *Magea* and *Xlr*, also failed to be expressed in cloned embryos even when the expression of *Xist* was controlled [10].

In this study, we used BIX as a specific inhibitor of G9a [27]. The H3K9me2 level at the *Magea2* promoter region was decreased by BIX treatment [26]. Consistent with this result, when we treated cloned embryos with BIX, H3K9me2 and HP1α levels were significantly decreased and *Magea2* gene expression was improved, while the expression levels of *Magea5* and *Xlr5c* were not increased significantly. However, the birth rate of cloned mice was not increased at all. Surprisingly, the acH3K9 level decreased, so several other H3K9me-related genes might remain silenced in BIX-treated cloned embryos and thereby lead to embryo death. Because Xist knockout or knockdown did not change the expression patterns of *Magea* and *Xlr* family genes in cloned embryos [6,10], H3K9me2 might be a reprogramming-resistant region of the SCNT nucleus, and other approaches targeting these sites might be needed for proper genomic reprogramming.

In conclusion, while BIX treatment did not increase the birth rate of cloned mice even though some epigenetic errors were corrected to some extent, LatA treatment promoted normal chromosome segregation in the context of proper F-actin formation and improved the full-term development of cloned embryos with epigenetic abnormalities remained. Thus, non-epigenetic factors, such as F-actin formation and chromosome segregation in early stage of preimplantation embryos, also play an important role in determining the success rate of mouse cloning. 
